# Chronic endometritis and recurrent reproductive failure: a systematic review and meta-analysis

**DOI:** 10.3389/fimmu.2024.1427454

**Published:** 2024-08-16

**Authors:** Carlo Ticconi, Annalisa Inversetti, Serena Marraffa, Luisa Campagnolo, Jephtah Arthur, Enrica Zambella, Nicoletta Di Simone

**Affiliations:** ^1^ Department of Surgical Sciences, Section of Gynecology and Obstetrics, University of Rome Tor Vergata, Rome, Italy; ^2^ Department of Biomedical Sciences, Humanitas University, Milan, Italy; ^3^ Istituto di Ricerca e Cura a Carattere Scientifico (IRCCS) Humanitas Research Hospital, Milan, Italy; ^4^ Department of Biomedicine and Prevention, University of Rome Tor Vergata, Rome, Italy

**Keywords:** chronic endometritis, recurrent pregnancy loss, recurrent implantation failure, infertility, reproductive failure chronic endometritis, reproductive failure, systematic review, meta-analysis

## Abstract

**Background:**

The endometrium holds a crucial role in reproduction by supporting blastocyst adhesion, cytotrophoblast invasion and fetal development. Among the various uterine disorders, endometritis, particularly chronic endometritis (CE), has gained attention due to its association with adverse reproductive outcomes (recurrent pregnancy loss (RPL), recurrent implantation failure (RIF), and infertility). The association between CE and adverse reproductive outcomes stresses the necessity for comprehensive diagnostic and therapeutic strategies to optimize fertility outcomes and support individuals in their journey towards parenthood.

**Aim:**

To explore the relationship between CE and reproductive disorders.

**Methods:**

Following PRISMA guidelines, a systematic review and meta-analysis using published data from 1990 to 2024 were carried out.

**Results:**

A population of 1,038 women was included. Regarding CE-infertility association, a positive correlation was found, with 19.46% CE rate in infertile women compared to 7.7% in controls (OR: 2.96, 95% CI 1.53-5.72, p 0.001). No significant association was observed between RIF and CE (OR: 1.10, 95% CI 0.26-4.61, p 0.90), CE rates in both groups were relatively comparable, with 6.35% in women with RIF and 5.8% in controls. On the opposite, a strong association between CE and RPL was found, reporting a CE rate of 37.6% in RPL cases compared to 16.4% in controls (OR: 3.59, 95% CI 2.46-5.24, p < 0.00001).

**Conclusions:**

CE appears to be associated to infertility and RPL, while no significant association was noted in cases of RIF.

**Systematic review registration:**

https://www.crd.york.ac.uk/prospero/#recordDetails PROSPERO, identifier CRD42024541879.

## 1 Introduction

Chronic endometritis (CE) is a persistent and mild inflammation of the endometrial mucosa. Currently there is no standardized or accepted definition of chronic endometritis, but the presence of numerous plasma cells in the stroma is the most sensitive and specific finding for the definition and diagnosis of this disease (1). This immunological alteration is believed to be a consequence of a bacterial infection. CE has been found to be associated with reproductive failure. However, there are still no clear recommendations on whether its inclusion in the primary work-up of infertile couples is essential.

While acute endometritis typically occurs in response to infection following childbirth, miscarriage, or certain medical procedures, chronic endometritis involves persistent inflammation that may go unnoticed for extended periods. The diagnosis of chronic endometritis requires a thorough strategy that incorporates clinical assessment, imaging investigations, and histopathological examination. Recent progressions in diagnostic methodologies have enhanced detection precision, underscoring the importance of maintaining a vigilant stance, especially among individuals encountering infertility or recurrent pregnancy losses (2).

Previous studies have underscored the correlation between chronic endometritis and unfavorable reproductive outcomes, resulting in reduced pregnancy rates compared to individuals without the condition, following assisted reproductive technologies (ART) interventions (3).

Some investigators have shown possible adverse effects of CE on human reproduction. The frequency of CE is 2.8–56.8% in infertility, 14–67.5% in recurrent implantation failure (RIF), and 9.3–67.6% in recurrent pregnancy loss (RPL) (4). The current epidemiological data exhibit significant heterogeneity.

A recent study by Volodarsky-Perel et al. highlights a significant association between chronic endometritis (CE) and infertility, particularly in women with endometrial polyps (EP) and a history of infertility. Vascular changes observed in the endometrium of infertile women suggest a potential link between CE and infertility-related vascular pathology (5).

The success of *in vitro* fertilization (IVF) has improved dramatically since its inception. However, there are still transfers that do not result in implantation. RIF presents a challenging development in assisted reproductive technology (ART) where despite multiple transfers, successful implantation does not occur (6). With the increasing success of *in vitro* fertilization, the demand for better outcomes has grown among patients and providers, leading to a rise in literature exploring recurrent implantation failure. Yet, there remains a lack of consensus on its definition. It can therefore be described as three failed IVF or ICSI (intracytoplasmic sperm injection) treatments, each with at least one fresh good quality embryo per transfer, or failure to achieve pregnancy after transfer of 10 good quality embryos (7).

A study investigated the prevalence of chronic endometritis in women with failed implantation, and its impact on subsequent live birth rates (LBRs) after antibiotic treatment (8). Chronic endometritis was found in 9% of participants, suggesting the inclusion of endometrial biopsy in evaluations. Those with chronic endometritis had more failed implantations and showed greater improvement in subsequent LBR after treatment.

Furthermore, mounting evidence suggests that chronic endometrial inflammation may disrupt the delicate balance necessary for successful implantation and pregnancy maintenance, thereby predisposing women to recurrent miscarriages (9, 10). Despite growing interest in this area (11), a comprehensive synthesis of available evidence is warranted to elucidate the nature of the relationship between CE and RPL.

Overall, the findings stress on the significance of the endometrial environment in embryo implantation and fetal development, urging further research and attention to CE’s role in reproductive health to optimize fertility outcomes and support individuals in their journey towards parenthood (12).

This systematic review and meta-analysis aimed to investigate the potential link between chronic endometritis (CE) and various clinically significant female reproductive disorders, such as infertility, RIF and RPL.

## 2 Materials and methods

This systematic review and meta-analysis were conducted and reported following the guidelines of the *Preferred Reporting Items for Systematic Reviews and Meta-Analyses* (PRISMA) (13).

As the review was based on data already published in the literature, approval from the territorial ethics committee was not required. The study protocol is currently undergoing evaluation in the *International Prospective Register of Systematic Reviews (PROSPERO registry)* with the ID CRD42024541879.

### 2.1 Inclusion and exclusion criteria

All studies examining the correlation between chronic endometritis (CE) and recurrent pregnancy loss (RPL), infertility, and repeated implantation failure (RIF) were included in the review. However, due to the absence of standardized diagnostic criteria for chronic endometritis and the varying definitions across the included studies, CE was identified based on the specific parameters outlined in each article reviewed.

Randomized controlled trials, cohort studies, case-control studies, and cross-sectional studies were deemed suitable for scientific analysis. The research and selection criteria were restricted to articles published in English from 1990 onwards.

Systematic reviews, meta-analyses, reviews, case reports, notes or letters, book chapters, errata, and conference abstracts were excluded. Only studies involving human participants were included, while those involving animals were excluded.

### 2.2 Sources of information

We conducted a systematic literature search spanning from January 1, 1990, to February 2, 2024, across five electronic databases*: PubMed, Scopus, Web of Science, ScienceDirect, and the Cochrane Library.*

### 2.3 Research strategy

For our research, we devised combinations of relevant Medical Subject Headings (MeSH) terms and keywords pertaining to the exposure and event under scrutiny. We crafted two distinct search strings for each of the five databases considered: one concentrating on terms related to chronic endometritis and recurrent pregnancy losses/spontaneous abortions, and another aimed at locating articles concerning chronic endometritis in connection with infertility and repeated implantation failure. This approach was chosen to prevent the creation of overly complex search strings, which might compromise the overall effectiveness of the search. Moreover, certain databases, such as *ScienceDirect*, have a maximum limit of eight logical or Boolean operators per search. Therefore, dividing the search into multiple parts enabled us to manage this limitation more effectively and ensure comprehensive coverage of the topics of interest. Additionally, we meticulously scrutinized the bibliographic lists of all identified articles to prevent the omission of pertinent data.

The following are the two search strings used for each database:

#### 2.3.1 Pubmed

1. (“Endometritis”[Mesh] OR endometritis*[tiab] OR endomyometritis OR endometrial inflammation [tiab] OR “CD138 antigen”[Mesh] OR CD138 antigen [tiab] OR “Plasma cells”[Mesh] OR plasma cell* [tiab] OR plasmacyte* [tiab]) AND (“Recurrent pregnancy loss”[Mesh] OR recurrent pregnancy loss*[tiab] OR recurrent abortion*[tiab] OR recurrent miscarriage*[tiab] OR recurrent early pregnancy loss*[tiab] OR “Abortion,habitual”[Mesh] OR abortion,habitual[tiab])
2. (“Endometritis”[Mesh] OR endometritis*[tiab] OR endomyometritis OR endometrial inflammation [tiab] OR “CD138 antigen”[Mesh] OR CD138 antigen [tiab] OR “Plasma cells”[Mesh] OR plasma cell* [tiab] OR plasmacyte* [tiab]) AND (“Infertility”[Mesh] OR infertility[tiab] OR sterility, reproductive[tiab] OR sterility[tiab] OR reproductive sterility[tiab] OR subfertility[tiab] OR sub-fertility[tiab] OR “Reproductive Techniques, Assisted”[Mesh] OR reproductive technique*, assisted[tiab] OR assisted reproductive technique*[tiab] OR technique*, assisted reproductive[tiab] OR assisted reproductive technic*[tiab] OR reproductive technic*, assisted[tiab] OR technic, assisted reproductive[tiab] OR reproductive technolog*, assisted[tiab] OR assisted reproductive technolog*[tiab] OR reproductive technolog*, assisted[tiab] OR technolog*, assisted reproductive[tiab] OR “Fertilization in Vitro”[Mesh] OR fertilization *in vitro* OR *in vitro* fertilization*[tiab] OR “Sperm Injection, Intracytoplasmic”[Mesh] OR sperm injection, intracytoplasmic[tiab] OR injection*, intracytoplasmic sperm[tiab] OR intracytoplasmic sperm injection*[tiab] OR ICSI[tiab] OR recurrent implantation failure[tiab] OR repeated implantation failure[tiab] OR implantation failure[tiab])

#### 2.3.2 Scopus

1. ((TITLE-ABS-KEY (“recurrent miscarriage”) OR TITLE-ABS-KEY (abortions) OR TITLE-ABS-KEY (“recurrent pregnancy loss”))) AND ((TITLE-ABS-KEY (endometritis) OR TITLE-ABS-KEY (endomyometritis) OR TITLE-ABS-KEY (“endometrial inflammation”) OR TITLE-ABS-KEY (“cd138 antigen”) OR TITLE-ABS-KEY (“plasma cells”) OR TITLE-ABS-KEY (“chronic endometritis”)))
2. ((TITLE-ABS-KEY (endometritis) OR TITLE-ABS-KEY (endomyometritis) OR TITLE-ABS-KEY (“endometrial inflammation”) OR TITLE-ABS-KEY (“CD138 antigen”) OR TITLE-ABS-KEY (“plasma cells”) OR TITLE-ABS-KEY (“chronic endometritis”)) AND ((TITLE-ABS-KEY (infertility) OR TITLE-ABS-KEY (sterility) OR TITLE-ABS-KEY (“reproductive sterility”) OR TITLE-ABS-KEY (“sub-fertility”) OR TITLE-ABS-KEY (“Reproductive Techniques Assisted”) OR TITLE-ABS-KEY (“recurrent implantation failure”) OR TITLE-ABS-KEY (“fertilization *in vitro*”))

#### 2.3.3 Web of Science

1. ((((((TS=(endometritis)) OR TS=(endomyometritis)) OR TS=(“endometrial inflammation”)) OR TS=(CD138)) OR TS=(“CD138 antigen”)) OR TS=(“chronic endometritis”)) OR TS=(“plasma cells”) AND ((((TS=(“recurrent pregnancy loss”)) OR TS=(“recurrent miscarriage”)) OR TS=(abortions)) OR TS=(“recurrent spontaneous abortion”))
2) ((((((TS=(endometritis)) OR TS=(endomyometritis)) OR TS=(“endometrial inflammation”)) OR TS=(CD138)) OR TS=(“CD138 antigen”)) OR TS=(“chronic endometritis”)) OR TS=(“plasma cells”) AND (((((((((TS=(“recurrent implantation failure”)) OR TS=(“repeated implantation failure”)) OR TS=(infertility)) OR TS=(sterility)) OR TS=(“reproductive sterility”)) OR TS=(sub-fertility)) OR TS=(“Reproductive Techniques Assisted”)) OR TS=(“Fertilization in Vitro”) OR TS=(“reproductive failure”)) OR TS= (RIF))

#### 2.3.4 ScienceDirect

1) [(“Chronic endometritis”) OR (“CD138 antigen”) OR (endometritis) OR (“plasma cells”) OR (“endometrial inflammation”)] AND [(“recurrent pregnancy loss”) OR (“recurrent miscarriage”) OR (“recurrent spontaneous abortion”) OR (abortions)]
2) [(Chronic endometritis)” OR (“CD138 antigen”) OR (“plasma cells”) OR (endometritis) OR (“endometrial inflammation”)] AND [(“recurrent implantation failure”) OR (“fertilization *in vitro*”)) OR (infertility) OR (“reproductive failure”)]

#### 2.3.5 Cochrane Library

1) (“Chronic endometritis” OR “CD138 antigen” OR “plasma cells” OR “endometritis” OR “endometrial inflammation”) AND (“recurrent pregnancy loss” OR “recurrent miscarriage” OR “recurrent spontaneous abortion” OR “abortions”)
2) (“Chronic endometritis” OR “CD138 antigen” OR “endometritis” OR “plasma cells” OR “endometrial inflammation”) AND (“recurrent implantation failure” OR “fertilization *in vitro*” OR “infertility” OR “reproductive failure”)

### 2.4 Inclusion criteria used for the selection of control patients

The general inclusion criteria used for the selection of control patients are summarized below:

1. Normal personal general and gynecologic history:
2. Regular menstrual cycles/Regular endocrine profile
3. Normal previous obstetric history

The detailed inclusion criteria for each selected study have been reported in the results section

### 2.5 Data selection and extraction process

Two authors (S.M. and C.T.) independently conducted electronic searches and analyzed bibliographic lists. Subsequently, they evaluated titles, abstracts, and full texts based on the pre-defined inclusion and exclusion criteria. Any discrepancies between the reviewers were resolved through mutual consensus or with the supervision of N.D.S.

The selected documents were then retrieved and thoroughly analyzed to extract the following information: first author’s name, publication year, country of origin, study type, duration if specified, participant characteristics, chronic endometritis diagnosis method, chronic endometritis definition, and the primary findings of each study.

### 2.6 Study outcomes

The present meta-analysis was conducted to evaluate the association between chronic endometritis and three specific female reproductive diseases. In particular, the aspects investigated are:

**-** Association of chronic endometritis and infertility
**-** Association of chronic endometritis and recurrent implantation failure (RIF)
**-** Association of chronic endometritis and recurrent pregnancy losses (RPL)

### 2.7 Study bias risk assessment

Three authors (S.M., C.T. and J.A.) utilized the Newcastle-Ottawa Scale (NOS) to assess the quality of the included studies, specifically focusing on non-randomized trials (case-control and cohort) (14). The quality assessment covered three domains: study group selection, group comparability, and assessment of exposure or outcome of interest, for case-control or cohort studies, respectively. Any discrepancies between the reviewers were resolved through discussion with a third-party auditor (A.I.).

The overall score ranged from 0 to 9, with the study considered qualitatively adequate if the total score was greater than or equal to 5. Additionally, funnel plots were generated for each result to examine potential publication bias.

### 2.8 Statistical analysis

Quantitative analysis of the extracted data was performed using RevMan 5.4 software. Study outcomes were presented using odds ratios (OR) with 95% confidence intervals (95% CI). A p value <0.05 indicated a statistically significant difference in results.

Heterogeneity among studies was assessed using I² statistics. The degree of Heterogeneity was classified as low if I² was less than 30%, moderate if between 30% and 70%, and high if greater than 70%. These criteria were set according to Higgins et al. (14) and Ioannidis (15).

The subsequent step involved determining the appropriate statistical model for the meta-analysis. If the heterogeneity index (I²) exceeded 70%, suggesting substantial variability among studies, the data were not combined for meta-analysis. If I² fell between 30% and 70%, indicating moderate heterogeneity, a random-effects model was chosen. Conversely, in cases where heterogeneity was less than 30%, a fixed-effects model was employed.

## 3 Results

### 3.1 Selection of studies

Following the research strategy, a total of 3,004 relevant publications were identified (*PubMed*: 522, *Scopus*: 1,447, *Web of Science*: 692, *ScienceDirect*: 230, *Cochrane Library*: 113). Upon removal of duplicates, the titles and abstracts of the remaining 1,511 documents were reviewed. No automated software was utilized for duplicate removal. Out of these 1,511 documents, 1,495 were excluded based on the imposed exclusion criteria, while 16 studies were initially selected for inclusion. Subsequently, after a thorough examination of the full texts, seven studies were excluded for various reasons: three lacked a control group consisting of healthy fertile women, in two, the presence or absence of chronic endometritis was a prerequisite for group selection, one did not report the prevalence of chronic endometritis in controls, and one did not aim to detect the prevalence of chronic endometritis.

**Figure 1:**
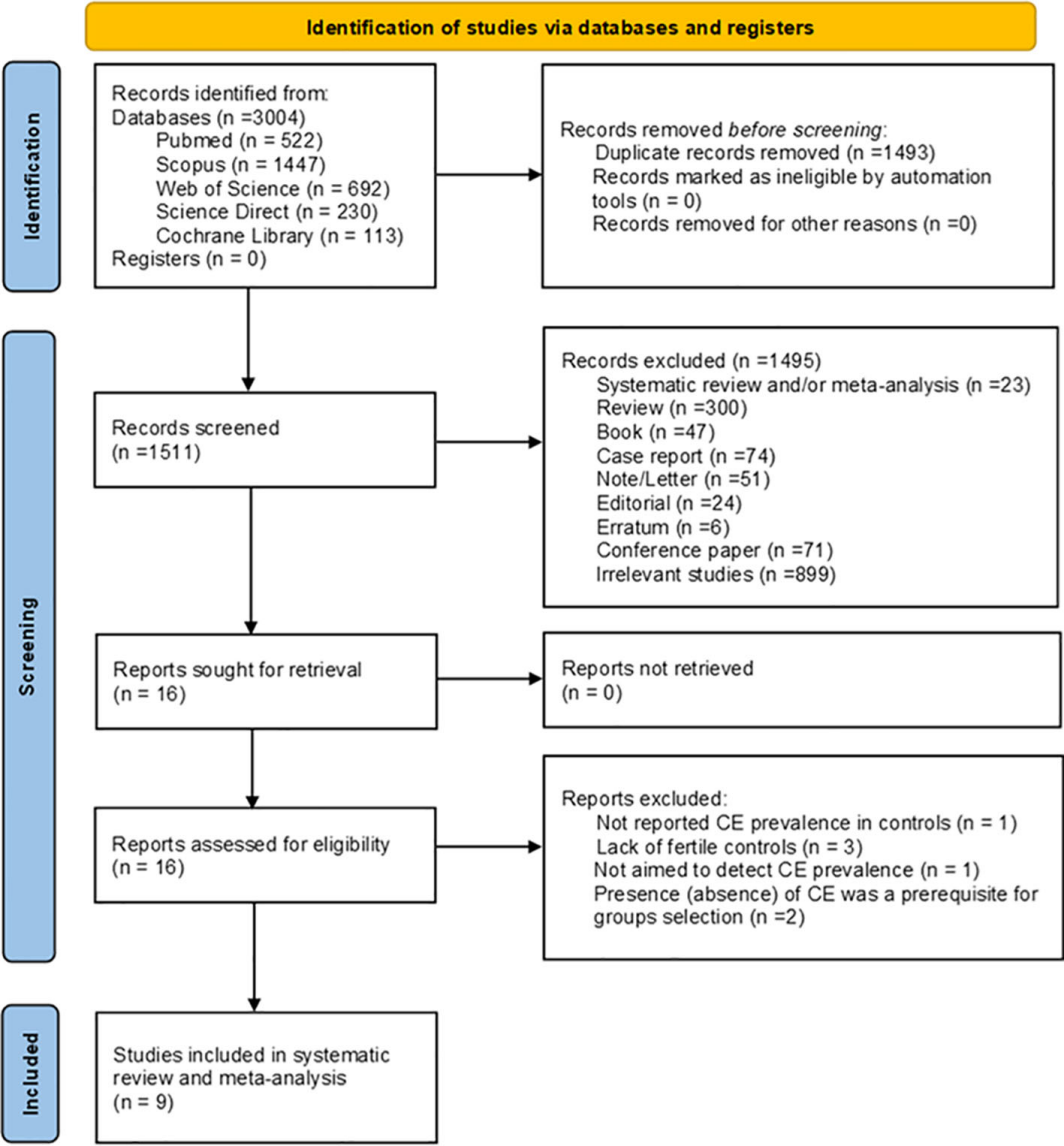
PRISMA 2020 Flowchart for bibliographic research.

Ultimately, nine studies were incorporated into the current systematic review and meta-analysis (5, 12, 16–23). **Figure 1** displays the PRISMA 2020 flowchart for systematic reviews.

It is important to highlight that despite generating two search strings for each database, a singular overall flowchart was devised. This was essential to ensure the exclusion of all duplicate.

### 3.2 Features of included studies

**Table 1** provides comprehensive details on the characteristics of all included studies. In terms of study design, the selection comprised four prospective cohort studies, one prospective and retrospective cohort study, two retrospective cohort studies, and two case-control studies.

**Table 1:** Main features of the included studies.

Study ID years and country	Study design and time of realization	Participant information		Diagnosis method of CE	CE definition	Main findings
		Group	Main characteristics			
Takimoto K et al. (20), Japan	Prospective cohort, from March 2021 to January 2023	Group A:24 RIF Group B: 27 RPL Group C: 29 fertile control women	RIF group: women with failure to achieve a clinical pregnancy after 2 or more transfer cycles, mean age 40 y/o, one had endometriosis, 2 PCOS, none had uterine myoma and received probiotics/prebiotics,4 received antibiotic treatment RPL group: women with 2 or more recurrent pregnancy losses, mean age 37 y/o, none had uterine myoma and received probiotics/prebiotics Fertile control group: women <44 y/o, regular menstrual cycles, without history of infertility, RPL, myoma, endometriosis, adenomyosis	IHC staining of the plasma cell marker CD138	Liu’s method: Plasma cell count >5,15/10 mm² Mc Queen DB score: 0: none/<1plasma cell/HPF(x40) 1: 1-5/HPF or cluster < 20cells 2:6-20/HPF or cluster>20 cells 3: ≥ 20/HPF or sheets of cells	The frequency of CE (plasma cells > 5.15/10 mm2) was higher in women with RPL (29.6%) than in fertile controls (6.8%, p < 0.05) The plasma cell count/10 mm2 in women with RPL (median 1.53, range 0–252.6, p < 0.01) and women with RIF (median 0.6, range 0–6.98, p < 0.05) was higher than in fertile controls (median 0, range 0–29) The relative dominance rate of *Lactobacillus iners* (median 4.7% vs. median 0%) and the positive rates of *Ureaplasma* species (36.3% vs. 8.6%) were higher in women with CE than in women without CE
Goto T et al. (19), Japan	Retrospective cohort, from January 2011 to March 2019	Group A: 49 RPL Group B: 17 controls	RPL group: women with 2 or more unexplained pregnancy losses, divided in euploid (n=22, mean age 31,40 y/o) and aneuploid miscarriage (n=27, mean age 33,70 y/o) Fertile control group: women with mean age 33,3 y/o, women who underwent an artificial abortion in the first trimester of pregnancy	IHC staining of the plasma cell marker CD138	Grade 1: 1 plasma cell/HPF Grade 2: ≥ 2 plasma cells/HPF	The prevalence of Grade 1 was 18.2% (4/22) in patients with euploid miscarriage, 37.0% (10/27) in patients with aneuploid miscarriage and 23.5% (4/17) in control women. The prevalence of Grade 2 was 45.5% (10/22) in patients with euploid miscarriage, 55.6% (15/27) in patients with aneuploid miscarriage and 23.5% (4/17) in control women. There was a significant difference in the prevalence of CD (p = 0.015). The LBR of patients with CD was similar to that of patients without CD
McQueen DB et al. (23), USA	Prospective and retrospective cohort, from February 2016 to February 2020	Group A: 50 RPL Group B: 26 controls	RPL group: women with 2 or more pregnancy losses, mean age 35,2 y/o, TSH<4mU/L, negative antiphospholipid antibodies, normal uterine anatomy Fertile control group: women with mean age 33,2 y/o, no history of RPL, infertility, uterine fibroids, polyps, PID or retained pregnancy tissue	H&E staining and IHC staining of the plasma cell marker CD138	H&E/CD138: ≥ 1, ≥2 and ≥5 plasma cells/10HPF with and without endometrial stromal changes	The results confirm increased sensitivity of CD138 staining compared to H&E. With CD138 staining, the prevalence of CE was 31% in controls and 56% in women with RPL No control had both plasma cells and endometrial stromal changes in biopsy samples compared to women with RPL (0% vs 30%)
Chiokadze M et al. (21), Germany, Georgia	Case-control, no data reported	Group A: 61 RPL Group B: 10 controls	RPL group: women with 2 or more consecutive pregnancy losses, mean age 33,5 y/o Control fertile group: women with mean age 27 y/o, no miscarriage, least one live birth, no autoimmune disease, antibiotic therapy, hormonal treatment or vaccination for at least 3 months	IHC staining of the plasma cell marker CD138	<3 CD138+ plasma cells/10 mm² Plasma cell count>5,15/10 mm² (Liu’s method)	The prevalence of CE (CD138+ <3 plasma cells/10mm²) was 20 % in controls and 22 % in women with RPL. With Liu’s method the potential influence of this condition was excluded The mean number of CD16+ cells was significantly increased in the endometrium of uRPL patients compared to controls (p < 0.001). No differences were observed in the mean values of CD45 (p = 0.06), CD56 (p = 0.99), and CD57 (p = 0.14). By additional analysis of these markers showed their different distributions in uRPL patients (p < 0.001 for CD45, CD56, and CD16; p = 0.003 for CD57) compared to controls
Volodarsky-Perel A et al. (2019), Canada (5)	Retrospective cohort, from 2015 to 2018	Group A: 137 infertile women Group B: 140 controls	Patients undergoing hysteroscopic polipectomy Infertile group: women with mean age 38 y/o, fallopian tubes patency, regular ovulatory cycles and normal partner’s sperm parameters Control fertile group: women with mean age 40 y/o no history of infertility, no hormone treatment in the previous 3 months before hysteroscopy, no spontaneous pregnancy in the previous 3 years before the procedure	IHC staining of the plasma cell marker CD138	Plasma cells >1/10HPF	The prevalence of CE in the group of infertile women was significantly higher than that in the control group (22.6% vs. 8.6%; p = 001) Women with primary infertility and those with secondary infertility showed no difference in CE prevalence Pregnancy outcome in infertile women with treated CE was like those who were infertile and without CE
Barath SH et al. (2019), (17)	Case-control, no data reported	Group A: 40 RPL Group B: 60 controls	Patients undergoing hysteroscopic RPL group: women with 3 or more unexplained pregnancy losses, mean age 28,8 y/o Control fertile group: women with mean age 32,8 y/o, vaginal bleeding or other causes except RPL and infertility, at least two non-aborted pregnancies whose last delivery was at least one year before study	H&E staining	Localized or diffuse endometrial bleeding symptoms and, in some cases, polyps<1mm in the endometrium	Endometritis rate was 8% in all patients. Patients with RPL had increased incidence of CE both hysteroscopically (30% vs. 6.7%; p<0.005) and pathologically (27.5% vs. 6.7%; p < 0.005)
Liu Y et al. (18), China	Prospective cohort, from December 2014 to June 2017	Group A: 39 RIF Group B: 93 RPL Group C: 48 infertility Group D: 40 controls	Mean age of all women 34,4 y/o RIF group: women with failure to achieve a clinical pregnancy after 3 or more transfer cycles RPL group: women with 3 or more consecutive unexplained pregnancy losses Infertile group: women undergoing endometrial scratch in a natural cycle preceding frozen-thawed embryo transfer Fertile control group: women who at least one live birth	H&E staining and IHC staining of plasma cell marker CD138	Methods: 1) CD138+ cell count/10HPF 2) CD138+/whole section 3) CD138+/unit area (cell density) Definition CE: presence CD138+ plasma cell count or density >95^th^ percentile	The use of CD138 staining was higher sensitive than H&E staining. The plasma cell count per unit area showed the least observer variability among the three methods and the prevalence of CE in women with RM, RIF, and Infertility were 10.8%, 7.7%, and 10.4%, respectively, in the controls was 5%
D'Ippolito S et al. (22), Italy	Prospective cohort, no data reported	Group A:27 RPL Group B:10 controls	Inclusion criteria for both groups: age ≤39 years, healthy, regular ovulatory cycles, normal endocrine profile, absence of abnormal ultrasonographic features, no use of any contraceptive drugs or intrauterine device (IUD) in the past 6 months, no vaginal infections. RPL group: women with 3 or more consecutive unexplained pregnancy losses, mean age 36,3 y/o	H&E staining	Endometrial inflammatory component (weak or absent)	The expression of NALP-3 inflammasome and ASC protein is greater in the endometrium of women with RPL compared to controls, as is the activation of caspase-1 and levels of IL-1b and IL-18 A significant statistical difference in the inflammatory component of the endometrium was highlighted between the two groups (P<0.0001)
Zolghadri J et al. (12), Iran	Prospective cohort, from January 2006 to December 2008	Group A:142 RPL Group B: 154 controls	Patients undergoing hysteroscopic RPL group: women with mean age 30,1 y/o, divided in primary (≥ 3 loss pregnancies before 20 week of gestation) and secondary unexplained RPL (≥3 with at least one pregnancy beyond 20 week of gestation) Control fertile group: mean age 30,8 y/o, with two or more pregnancies with no history of pregnancy loss	H&E staining	Presence of plasma cells in endometrial stroma >1/HPF	RPL group had higher incidence of CE compared to controls both hysteroscopically (67.6% vs. 27.3%; p < 0.0001) and pathologically (42.9% vs. 18.2%; p < 0.0001) Patients with secondary RPL demonstrated a higher prevalence of CE both pathologically (83.9% vs. 45.9%; p < 0.0001) and hysteroscopically (58.1% vs. 24.6%; p < 0.0001)

#### 3.2.1 Population

In total, the study encompassed a population of 1,038 women, consisting of 185 experiencing infertility, 63 facing repeated implantation failures, 489 diagnosed with recurrent miscarriages, and 486 healthy fertile women serving as controls.

While the definition of infertility was not explicitly outlined, all sources aligned with the interpretation of the inability to achieve pregnancy after twelve months of regular, unprotected sexual intercourse. Repeated implantation failure was characterized as the inability to achieve a clinical pregnancy after two failed embryo transfer cycles according to Takimoto et al. (20), while Liu Y et al. (18) considered three or more failed transfers. Regarding recurrent miscarriage, definitions varied among the selected studies. Four studies defined it as two or more miscarriages (19–21, 23), while four others considered three or more miscarriages (12, 17, 18, 22).

The results of the inclusion criteria used for the selection of control patients in each specific study are the following:

1. Normal personal general and gynecologic history:
  - no known medical conditions (12)
  - no history of gynecological conditions (uterine myoma, adenomyosis, endometriosis, malignancy, or surgery requiring intrauterine manipulation after the last delivery (20), polyps, pelvic inflammatory disease, or retained pregnancy tissue (21) or absence of abnormal ovarian and endometrial ultrasonographic features (22)
  - no autoimmune disease (21)
2. Regular menstrual cycles/regular endocrine profile:
  regular menstrual cycles for ≥ 1 year (20) or regular endocrine profile/normal FSH serum levels on day 3 of the menstrual cycle (22)
3. Normal previous obstetric history:
  - no previous history or treatment of infertility or RPL (5, 12, 20, 23)
  - spontaneous pregnancy within the previous 3 years (5) or ≥ 2 pregnancies whose last child was conceived within the previous 1 year (12) or ≥ 2 pregnancies whose last delivery was ≥ 1 year before the study (17) or ≥1 live birth within the previous 2 years (18) or ≥1 normal delivery (20)
  - no history of preeclampsia or intrauterine growth retardation (12)

In both studies by Chiokadze and McQueen, endometrial samples were collected in patients undergoing oocyte cryopreservation for elective fertility preservation or egg donation (21, 23).

Obstetric inclusion criteria for selection controls patients were assessed in every studies.

#### 3.2.2 Diagnosis of CE

Out of the nine selected studies, four exclusively employ immunohistochemistry (IHC) for diagnosing chronic endometritis, utilizing the CD138 marker (syndecan-1) (5, 19–21). Meanwhile, three studies solely rely on conventional hematoxylin and eosin (H&E) staining (12, 17, 22). Two studies employ both diagnostic methodologies (18, 23). Furthermore, criteria for plasma cell counts vary among the studies due to the lack of international consensus on this matter.

### 3.3 Risk of bias in studies

Among the cohort studies, five scored 7 and two scored 8. Both case-control studies scored 5.

NOS assessment scores are shown in **Tables 2**, **3**.

**Table 2:** NOS scores in cohort studies.

Study	Selection				Comparability	Outcome			Total quality score
	Representativeness of the exposed cohort	Selection of the non exposed cohort	Ascertaiment of exposure	Demonstration that outcome of interest was not present at start of study	Comparability of cohorts on the basis of the design or analysis	Assessment of outcome	Follow-up long enough for outcomes to occur	Adequacy of follow up of cohorts	
Takimoto K et al. (20)	⋆	⋆	⋆	⋆	⋆⋆	–	⋆	⋆	8
Goto et al. (19)	–	⋆	⋆	⋆	⋆	⋆	⋆	⋆	7
McQueen DB et al. (23)	⋆	⋆		⋆	⋆	⋆	–	⋆	7
Volodarsky-Perel A et al. (2019)	⋆	⋆	⋆	⋆	⋆⋆	⋆	–	–	7
Liu Y et al. (18)	⋆	⋆	⋆	⋆	⋆	⋆	⋆	⋆	8
D'Ippolito S et al. (22)	⋆	⋆	⋆	⋆	⋆	⋆	–	⋆	7
Zolghadri J et al. (12)	⋆	⋆	⋆	⋆	⋆	⋆	–	⋆	7

The stars are the symbols commonly used in the evaluation of NOS scale.

**Table 3:** NOS score in a case-control study.

Study	Selection				Comparability	Exposure			Total quality score
	Adequacy of case definition	Representativeness of the cases	Selection of Controls	Definition of Controls	Comparability of cases and controls on the basis of the design or analysis	Ascertainment of exposure	Same method of ascertainment for cases and controls	Non- Response Rate	
*Chiokadze M et al.* (21)	⋆	⋆	–	⋆	⋆	⋆	–	–	5
*Barath SH et al. (2019) (17)*	–	–	⋆	⋆	⋆	⋆	⋆	–	5

The stars are the symbols commonly used in the evaluation of NOS scale.

### 3.4 Summary of results

**Figure 2:**
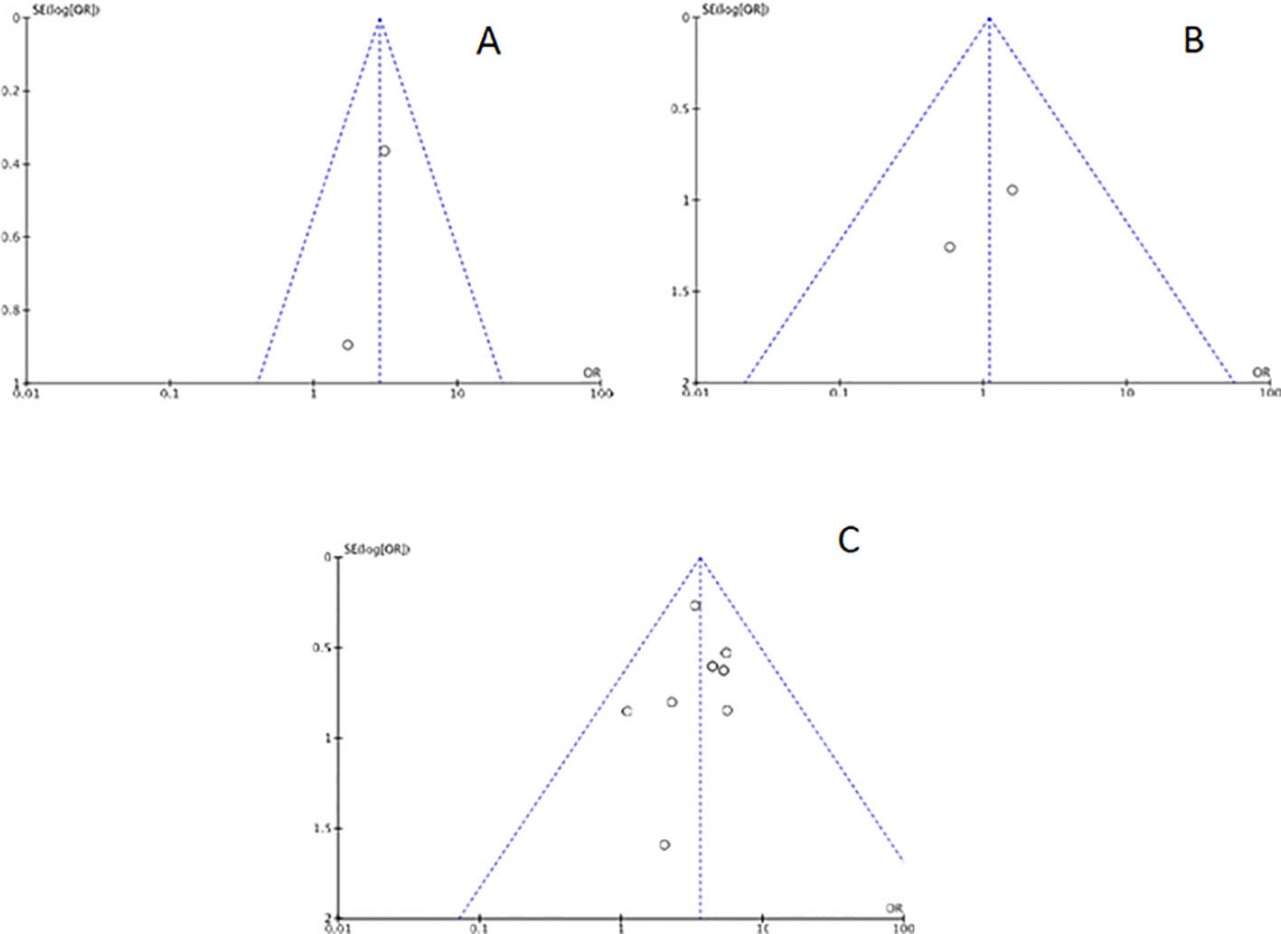
Funnel plot for each main outcome: **(A)** Chronic endometritis and infertility; **(B)** Chronic endometritis and Recurrent Implantation Failure; **(C)** Chronic endometritis and Recurrent Pregnancy Loss.

**Figure 3:**
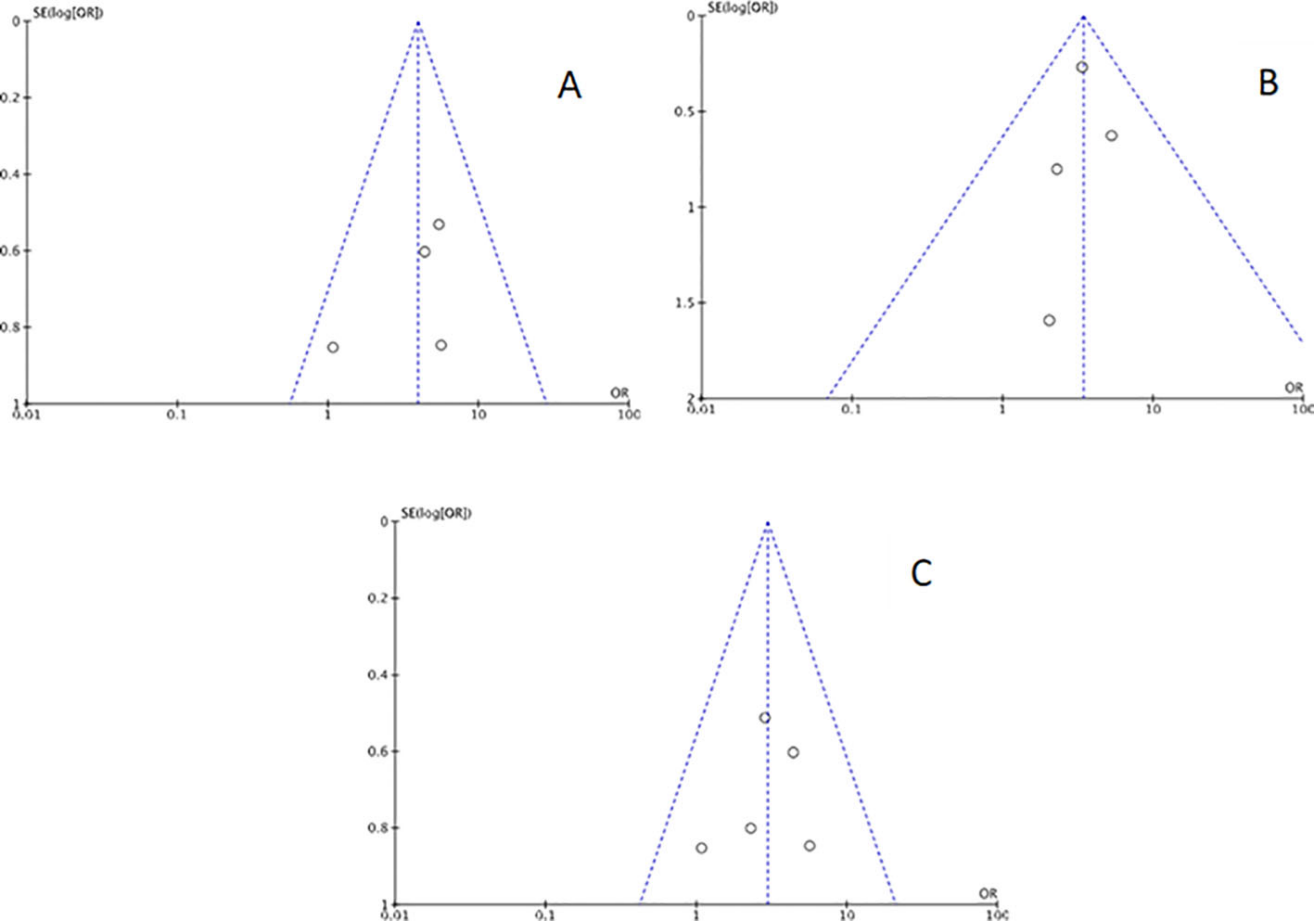
Funnel plot for sensitivity analysis: **(A)** studies with Chronic endometritis and Recurrent Pregnancy Loss defined as two or more abortions. **(B)** studies with Chronic endometritis and Recurrent Pregnancy Loss defined as three or more abortions. **(C)** studies with Chronic endometritis diagnosed with IHC for CD138 and RPL.

Funnel plots were created for each outcome to assess potential publication bias. Inspection of the funnel charts visually indicated no asymmetry (**Figures 2**, **3**).

#### 3.4.1 Chronic endometritis and infertility

A quantitative analysis of chronic endometritis prevalence in women with infertility was conducted based on two studies (5, 18). The study encompassed a total of 185 infertile women, including 36 with chronic endometritis, and 180 controls, including 14 with chronic endometritis.

**Figure 4:**
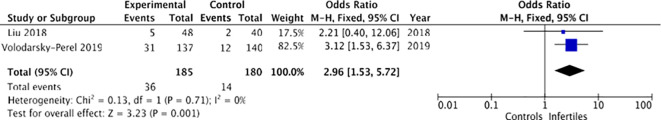
Forest plot for chronic endometritis and infertility.

Combined results from the two studies (5, 18) revealed a positive association between infertility and chronic endometritis: a higher prevalence of chronic endometritis was observed in infertile women (36/185 [19.46%]) compared to controls (14/180 [7.7%]). Utilizing a fixed-effects model, the odds ratio (OR) was calculated to be 2.96, with a 95% confidence interval (CI) of 1.53-5.72 and a p-value of 0.001. Heterogeneity was minimal (I² 0%) (**Figure 4**).

#### 3.4.2 Chronic endometritis and recurrent implantation failure

A quantitative analysis of chronic endometritis prevalence in patients with RIF was conducted based on two studies (18, 20). The study involved a total of 63 women with RIF, including 4 with chronic endometritis, and 69 controls, including 4 with chronic endometritis.

**Figure 5:**
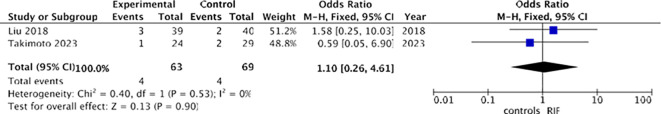
Forest plot for chronic endometritis and repeated implantation failure (RIF).

Combined results from the two studies (18, 20) revealed no significant association between recurrent implantation failure and chronic endometritis. Utilizing a fixed-effects model, the odds ratio (OR) was calculated to be 1.10, with a 95% confidence interval (CI) of 0.26-4.61 and a p-value of 0.90. Heterogeneity was negligible (I² = 0%) (**Figure 5**).

The prevalence of chronic endometritis in both groups was quite similar, with 6.35% in women with RIF and 5.8% in controls.

#### 3.4.3 Chronic endometritis and recurrent pregnancy loss

A quantitative analysis of chronic endometritis prevalence in women with recurrent pregnancy loss was conducted based on eight studies (12, 17–23). The study encompassed a total of 489 women with RPL, including 184 with chronic endometritis, and 346 controls, including 57 with chronic endometritis.

**Figure 6:**
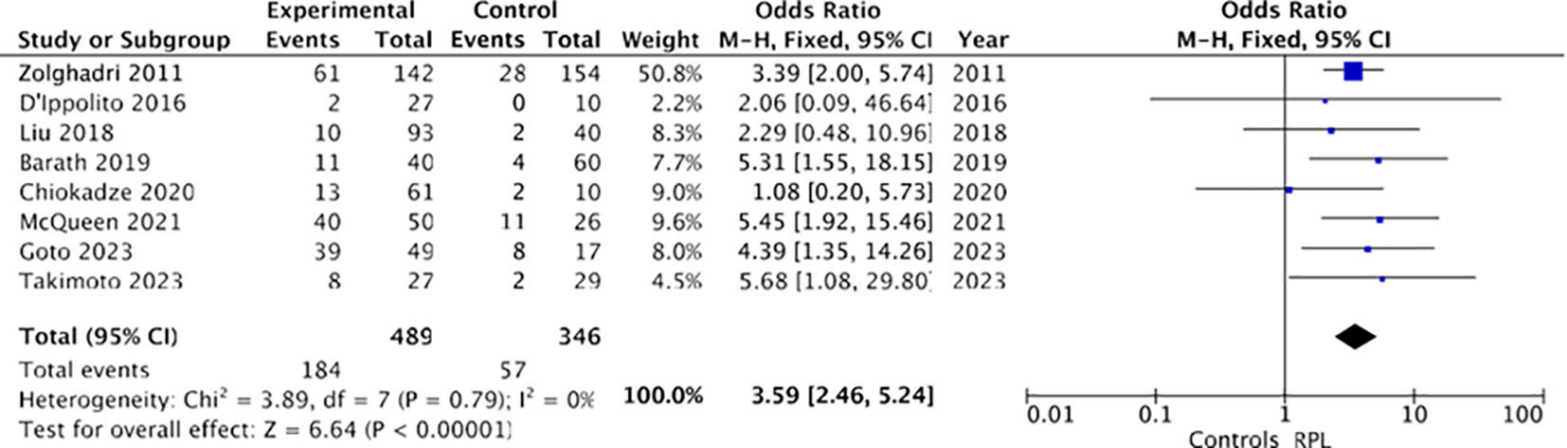
Forest plot for chronic endometritis and recurrent pregnancy loss (RPL).

Combined results from the eight studies revealed an association between recurrent miscarriage and chronic endometritis: a higher proportion of chronic endometritis was observed among women with RPL (184 out of 489 [37.6%]) compared to controls (57 out of 346 [16.4%]). Utilizing a fixed-effects model, the odds ratio (OR) was calculated to be 3.59, with a 95% confidence interval of 2.46-5.24 and a p-value of less than 0.00001. Heterogeneity was minimal (I²= 0%), as depicted in **Figure 6**.

### 3.5 Sensitivity analysis

**Figure 7:**
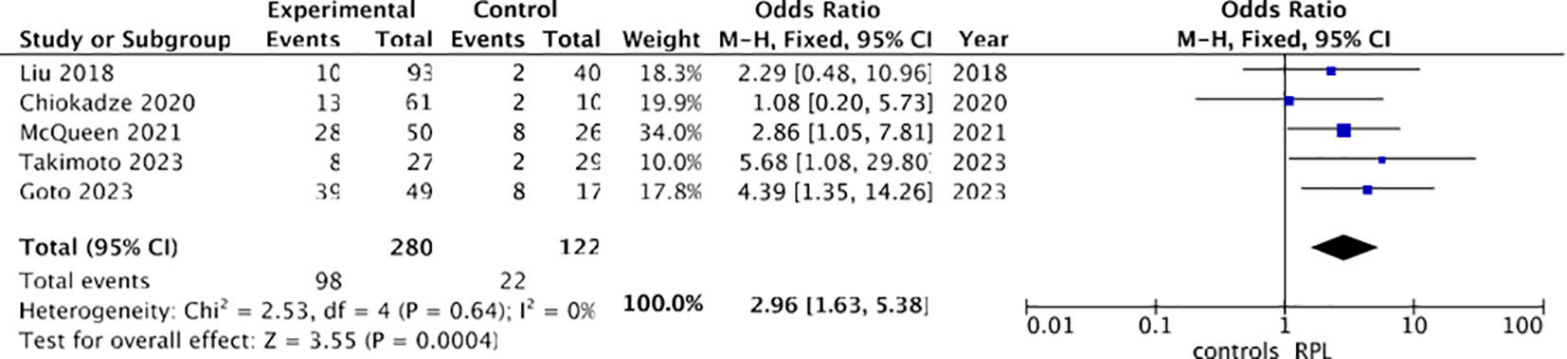
Forest plot for sensitivity analysis: CE diagnosed with IHC CD138 and RPL.

Three sensitivity analyses were conducted, all pertaining to RPL. In five out of the nine included studies (**Table 1**), immunohistochemistry (IHC) employing CD138 was utilized for chronic endometritis diagnosis. Consequently, we performed a sensitivity analysis incorporating only these five studies. As depicted in the forest plot in **Figure 7**, the findings closely resembled those of the previous analysis: a higher prevalence of chronic endometritis was observed among women with recurrent miscarriage (98/280 [35%]) compared to controls (22/122 [18%]). A fixed-effects model was consistently applied, given the absence of heterogeneity (I²=0%). The odds ratio (OR) was calculated to be 2.96, with a 95% confidence interval (CI) of 1.63-5.38 and a p-value of 0.0004.

The remaining two sensitivity analyses were performed based on the definition of recurrent miscarriage provided in the various included studies (**Table 1**).

**Figure 8:**
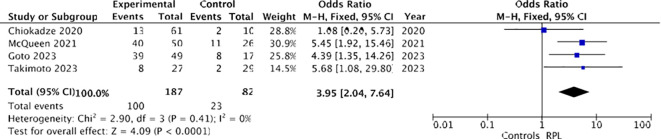
Forest plot for sensitivity analysis: CE and recurrent pregnancy loss defined as two or more spontaneous pregnancy losses.

Firstly, considering only those studies (17–19, 21) in which recurrent miscarriage was defined as two or more spontaneous pregnancy losses, a higher prevalence of chronic endometritis was observed among women with RPL (100/187 [53.4%]) compared to controls (23/82 [28%]). Utilizing a fixed-effects model, the odds ratio (OR) was calculated to be 3.95, with a 95% confidence interval (CI) of 2.04-7.64 and a p-value of less than 0.00001. Heterogeneity was absent (I² = 0%), as illustrated in **Figure 8**.

**Figure 9:**
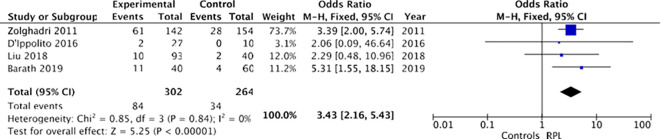
Forest plot for sensitivity analysis: CE and Recurrent pregnancy loss defined as three or more spontaneous pregnancy losses.

Similarly, restricting the sensitivity analysis to only those studies (12, 17, 18, 22) that defined RPL as three or more losses, a higher prevalence of chronic endometritis was observed among women with RPL (84/302 [27.8%]) compared to controls (34/264 [12.1%]). Utilizing a fixed-effects model, the odds ratio (OR) was calculated to be 3.43, with a 95% confidence interval (CI) of 2.16-5.43 and a p-value of less than 0.00001. Heterogeneity was also absent (I² = 0%), as depicted in **Figure 9**.

## 4 Discussion

Chronic endometritis, characterized by persistent inflammation of the endometrium (24), commonly arises from intrauterine infections attributed to bacteria such as *Escherichia coli, Enterococcus faecalis, Streptococcus, Staphylococcus*, as well as *Mycoplasma* and *Ureaplasma* species (3). Over the past two decades, there has been a burgeoning interest in researching endometrial diseases and their impact on reproductive health. However, determining the prevalence of CE among women of reproductive age remains challenging, primarily due to the difficulties associated with obtaining endometrial tissue samples from healthy women for biopsy. Moreover, chronic endometritis is asymptomatic in approximately 25% of cases, or it may present with nonspecific symptoms that may go unnoticed for extended periods (1).

Further complicating research in this area is the absence of a universally agreed-upon definition and standardized diagnostic criteria of CE. Nevertheless, experts concur that the presence of endometrial plasma stromal cells endometrial stromal plasma cells (ESPCs) represents the most specific and sensitive indicator of this disorder (25). Presently, the gold standard for diagnosis involves identifying plasma cells through immunohistochemical (IHC) staining targeting the CD138 marker (syndecan-1) (26), a method demonstrated to be more sensitive and precise than conventional hematoxylin-eosin (H&E) staining. However, there remains no consensus regarding the specific threshold of plasma cells necessary for definitively diagnosing chronic endometritis (27), which may impede the interpretation and comparability of findings across studies.

An alternative diagnostic approach involves hysteroscopic examination to identify endometrial characteristics indicative of chronic endometritis, including a strawberry appearance, focal hyperemia, micropolyps, stromal edema, and hemorrhagic spots (28). *McQueen et al.* suggested defining CE as the detection of one or more plasma cells per ten high-magnification fields (HPF), particularly in the presence of endometrial stromal alterations (29).

Recent research has highlighted the emergence of additional immunohistochemical markers like MUM-1 and advanced molecular biology techniques for identifying microbial species undetectable through classical microbiological culture (30, 31).

The aforementioned challenges, coupled with the lack of comprehensive clinical evidence in this area, render the assessment of chronic endometritis (CE) uncertain regarding its role in various critical reproductive pathologies, such as infertility, RIF and RPL. Consequently, given these uncertainties, current guidelines do not recommend routine endometrial biopsy for CE investigation (31, 32).

The current epidemiological data exhibit significant heterogeneity: the prevalence of chronic endometritis (CE) among women with infertility varies widely from 2.8% to 56.8%, while among those with repeated implantation failures (RIF) it ranges from 14% to 67.5%, and in cases of recurrent miscarriage, it spans from 9.3% to 67.6% (4).

Furthermore, recent systematic reviews and/or meta-analyses have primarily focused on assessing the impact of antibiotic therapy for CE on reproductive outcomes. Hence, this systematic review and meta-analysis were undertaken to address these gaps.

The findings from this study reveal that chronic endometritis (CE) is more prevalent in women experiencing infertility and recurrent miscarriages (RPL) compared to the control group, with rates of 19.46% versus 7.7% for infertility and 37.6% versus 16.4% for RPL. Conversely, no significant disparities in CE prevalence were observed between women with RIF and the control group. However, since the overall number of women included in the analysis of the effect of CE on RIF was limited to 132 subjects (63 women with RIF and 69 control women), further studies are needed before a definitive conclusion regarding the lack of association of CE with RIF can be drawn. Moreover, CE patients with RIF usually are given antibiotic treatment before embryo transfer. The therapy could affect the final outcome and the results.

One of the limitations of this study pertains to the small sample size of subjects available for comparison across each category of reproductive pathologies investigated, despite the extensive duration of the research (1990–2024) and the utilization of five electronic databases.

This underscores the aforementioned challenge of obtaining healthy endometrial tissue samples.

Moreover, the presence of divergent definitions of RPL, coupled with the absence of a universally accepted criterion for plasma cell counts and the utilization of varied diagnostic methods for chronic endometritis (CE), further complicates the harmonization of the overall data. In an effort to mitigate these challenges, sensitivity analyses were conducted, yielding results akin to those obtained through the primary analysis.

Specifically, in the sensitivity analysis incorporating only studies employing immunohistochemical (IHC) staining for CD138 in diagnosing chronic endometritis (CE), a higher proportion of CE was observed among women with recurrent pregnancy loss (RPL) compared to controls (35% vs. 18%). In the other two sensitivity analyses, studies were categorized based on the definition of recurrent miscarriage: either as two or more miscarriages or as three or more miscarriages. In both scenarios, a greater prevalence of CE was noted in women with RPL compared to controls (53.4% vs. 28% for RPL defined as two or more miscarriages, 27.8% vs. 12.1% for RPL with three or more pregnancy losses). The hypothesis that the presence of chronic endometritis (CE) may correlate with infertility and RPL holds biological plausibility. Chronic inflammation can disrupt the delicate immunological equilibrium within the endometrium during implantation and early pregnancy stages. Since it is reported that the expression of proinflammatory cytokines in the endometrium of women with history of RPL is upregulated compared with controls, D’Ippolito et al. (22) hypothesized a role for an abnormal aspecific activation of the proteic system infammosome. In detail, they showed that NALP-3/ASC inflammosome is expressed in human endometrium and, furthermore, it is increased in the endometrium obtained from women with history of RPL. Due to the lack of specificity of the innate immune system, several stimuli might be responsible of the inflammosome activation.

While previous studies have explored the pathophysiological mechanisms underlying this interference (9, 10), ongoing advancements in knowledge may unveil novel pathways. This area of research remains dynamic and continuously evolving. Moreover, this association finds support in studies investigating the positive effects of treatment rather than directly assessing the prevalence of chronic endometritis.

Due to the limited sample size, we are unable to provide precise percentages for both healthy individuals and those affected by these diseases. Further studies may yield significant variations in results.

Concerning RIF, insufficient available data hinder us from drawing definitive conclusions regarding the potential impact of chronic endometritis on this condition.

In summary, chronic endometritis may play a role in the development of certain significant reproductive failures, particularly infertility and RPL.

It is worth noting that, to the best of our knowledge, this study is the only meta-analysis addressing this issue by employing healthy and fertile women as a control group. In contrast, other meta-analyses in the literature have predominantly, if not exclusively, focused on comparing cases of RPL with cases of RIF.

## 5 Conclusions

The gathered data reaffirmed the existing scientific literature. It was observed that women experiencing infertility exhibited a notably higher prevalence of chronic endometritis compared to controls, with rates of 19.46% versus 7.7%, respectively (OR 2.96, p 0.001, I²=0%). Similarly, among women with RPL, a comparable pattern emerged, with a prevalence of CE at 37.6% versus 16.4% in controls (OR 3.59, p<0.00001, I²= 0%). Conversely, no significant association was found between CE and RIF, potentially due to the limited sample size analyzed. These findings contribute further evidence to support a potential correlation between CE and adverse reproductive outcomes. Nonetheless, definitive conclusions regarding the role of CE in women with reproductive disorders necessitate additional clinical investigations to elucidate its true impact on female reproductive health. Establishing an international consensus on diagnostic criteria is imperative to establish clear clinical guidelines for the diagnosis and management of CE in patients with reproductive disorders.

Ultimately, this review underscores the significance of CE within the spectrum of female reproductive pathologies and emphasizes the importance of its thorough assessment and management in clinical settings.

## Data Availability

The raw data supporting the conclusions of this article will be made available by the authors, without undue reservation.

## Glossary

**Table:**

*Medically Assisted Reproduction (MAR)*	Reproduction brought about through various interventions, procedures, surgeries, and technologies to treat different forms of fertility impairment and infertility. These include ovulation induction, ovarian stimulation, all ART procedures, uterine transplantation, and intra-uterine, intracervical, and intravaginal insemination with semen of husband/partner or donor.
*Assisted Reproductive Technologies (ART)*	All interventions that include the *in vitro* handling of both human oocytes and sperm or of embryos for the purpose of reproduction, such as *In Vitro* Fertilization (IVF). Fertility care Interventions that include support and fertility management with an intention to assist patients to realize their parenthood goals.
*In Vitro Fertilization (IVF)*	A sequence of procedures that involves extracorporeal fertilization of gametes. It includes conventional IVF and intracytoplasmic sperm injection (ICSI), a procedure in which a single spermatozoon is injected into the oocyte cytoplasm.
